# Prediction of 1-octanol solubilities using data from the Open Notebook Science Challenge

**DOI:** 10.1186/s13065-015-0131-2

**Published:** 2015-09-24

**Authors:** Michael A. Buonaiuto, Andrew S. I. D. Lang

**Affiliations:** Department of Computing and Mathematics, Oral Roberts University, 7777 S. Lewis Avenue, Tulsa, OK 74171 USA

**Keywords:** 1-Octanol solubility, Open notebook science, Modeling

## Abstract

**Background:**

1-Octanol solubility is important in a variety of applications involving pharmacology and environmental chemistry. Current models are linear in nature and often require foreknowledge of either melting point or aqueous solubility. Here we extend the range of applicability of 1-octanol solubility models by creating a random forest model that can predict 1-octanol solubilities directly from structure.

**Results:**

We created a random forest model using CDK descriptors that has an out-of-bag (OOB) R^2^ value of 0.66 and an OOB mean squared error of 0.34. The model has been deployed for general use as a Shiny application.

**Conclusion:**

The 1-octanol solubility model provides reasonably accurate predictions of the 1-octanol solubility of organic solutes directly from structure. The model was developed under Open Notebook Science conditions which makes it open, reproducible, and as useful as possible.

**Graphical abstract:**
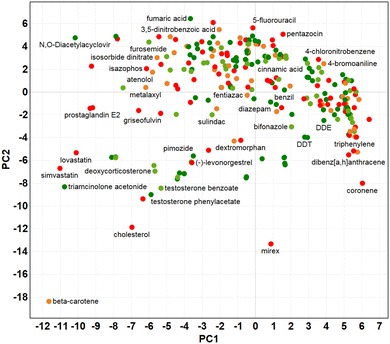
:

**Electronic supplementary material:**

The online version of this article (doi:10.1186/s13065-015-0131-2) contains supplementary material, which is available to authorized users.

## Background

The solubility of organic compounds in 1-octanol is important because of its direct relationship to the partition coefficient logP used in pharmacology and environmental chemistry. Current models that can be used to predict 1-octanol solubility include group contribution methods [1] and often include melting point as a descriptor [2–4]. The most recent model by Admire and Yalkowsky [4] gives a very useful rule of thumb to predict molar 1-octanol solubility from just the melting point

1 $${\text{Log S}}_{\text{oct}} = 0. 50 - 0.0 1\cdot \left( {{\text{mp}} - 2 5} \right),$$

where the compound melting point mp is in °C for compounds that are solid at room temperature and is taken to be 25 for liquids. Abraham and Acree [5] refined Admire and Yalkowsky’s model by appending the melting point term to their linear free energy relationship (LFER) model

2 $${\text{Log S}}_{\text{oct}} = {\text{c}} + {\text{e}} \cdot {\text{E}} + {\text{s}} \cdot {\text{S}} + {\text{a}} \cdot {\text{A}} + {\text{b}} \cdot {\text{B}} + {\text{v}} \cdot {\text{V}} + \lambda \cdot {\text{A}} \cdot {\text{B}} + \mu \cdot \left( {{\text{mp}} - 2 5} \right),$$

where E is the solute excess molar refractivity in units of (cm^3^/mol)/10, S is the solute dipolarity/polarizability, A and B are the overall or summation hydrogen bond acidity and basicity, and V is the McGowan characteristic volume in units of (cm^3^/mol)/100. The A·B term was added to deal with the solute–solute interactions. The coefficients were found using linear regression against the solubilities of solutes with known Abraham descriptors with the following result:

3 $$\begin{aligned} {\text{Log S}}_{\text{oct}} = 0. 4 80 - 0. 3 5 5\cdot {\text{E}} - 0. 20 3\cdot {\text{S}} + 1. 5 2 1\cdot {\text{A}} - 0. 40 8\cdot {\text{B}} + 0. 3 6 4\cdot {\text{V}} - 1. 2 9 4\cdot {\text{A}} \cdot {\text{B}} - 0.00 8 1 3\cdot \left( {{\text{mp}} - 2 5} \right) \hfill \\ {\text{N}} = 2 8 2,{\text{ SD}} = 0. 4 7,{\text{ Training Set R}}^{ 2} = 0. 8 30 \hfill \\ \end{aligned}$$

In the present study, we improve upon previous models by creating a nonlinear random forest model using solubility data from the Open Notebook Science Challenge [6], an open data, crowdsourcing research project that collects and measures the solubilities of organic compounds in organic solvents created by Jean-Claude Bradley and Cameron Neylon. The challenge is, in turn, part of Jean-Claude Bradley’s UsefulChem program, an open drug discovery project that uses open notebook science [7].

## Procedure

The 1-octanol solubility data in this paper were extracted from the Open Notebook Science Challenge solubility database [8]. We removed all items that were marked “DONOTUSE.” For compounds with multiple solubility values that included values listed in the Abraham and Acree paper, we kept only the solubility values that were listed in the Abraham and Acree paper. If no Abraham and Acree paper value was available, then we kept the Raevsky, Perlovich, and Schaper value instead. In the rare case that two Abraham and Acree (or Raevsky, Perlovich, and Schaper) paper values were listed for a single chemspider ID (CSID), we kept the higher of the two values.

**Fig. 1:**
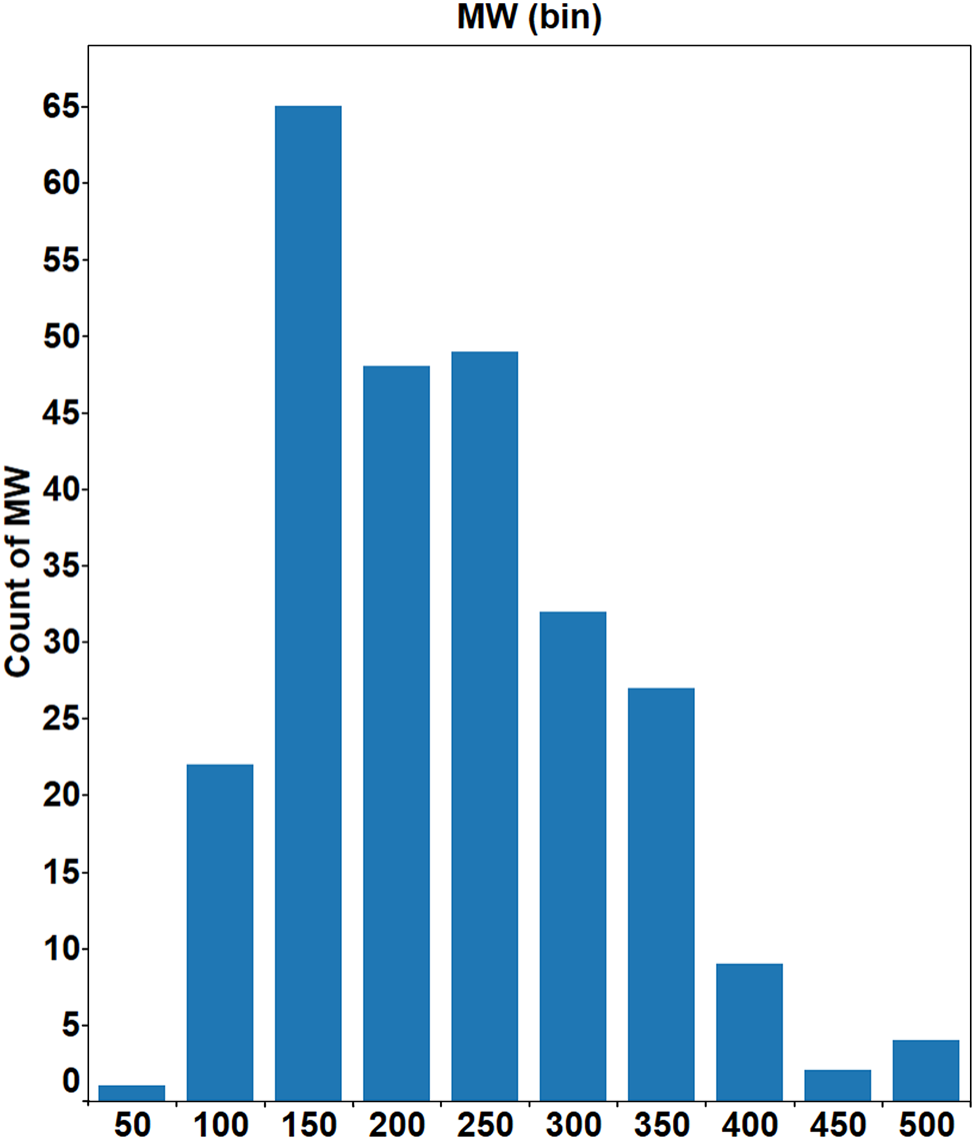
Mass distribution of the compounds in our study. 94 % of compounds have a molecular weight between 100 and 400 Da

**Fig. 2:**
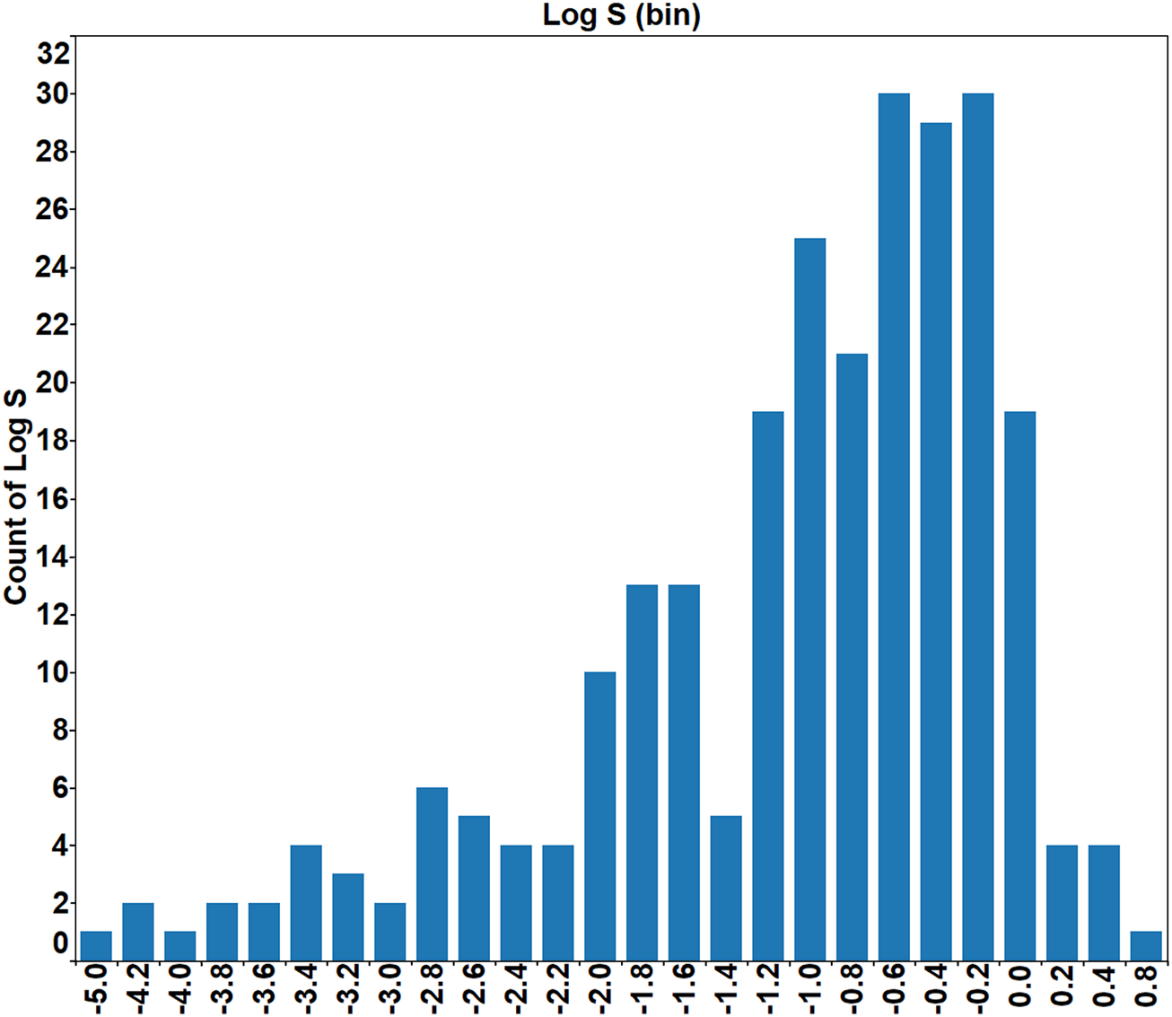
Solubility distribution of the compounds in our study. 76 % of compounds have solubility values between 0.01 and 1.00 M

**Fig. 3:**
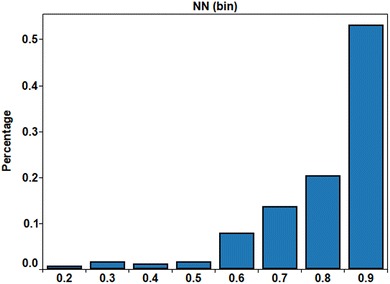
Nearest neighbor Tanimoto similarity

The collection and curation process left us with 261 data points to model, see Additional file 1. The structures in our dataset are not very diverse and can be characterized, in general, as relatively small organic compounds with 1-octanol solubility values between 0.01 and 1.00 M, see Figs. 1, 2, and 3.

Two features about the chemical space are immediately apparent. Firstly, the dataset has 50 carboxylic acids which is a common feature for both Abraham and Acree datasets and the Open Notebook Science Challenge dataset where the primary focus is on measuring solubilities for the same compound in several non-aqueous solvents. While common in non-aqueous solubility studies, sometimes one does have to consider dimerization for carboxylic acids [9]. Secondly, there are only 50 compounds that have a single Lipinski’s Rules failure (all the rest having zero failures), suggesting the dataset could be characterized as drug-like.

**Fig. 4:**
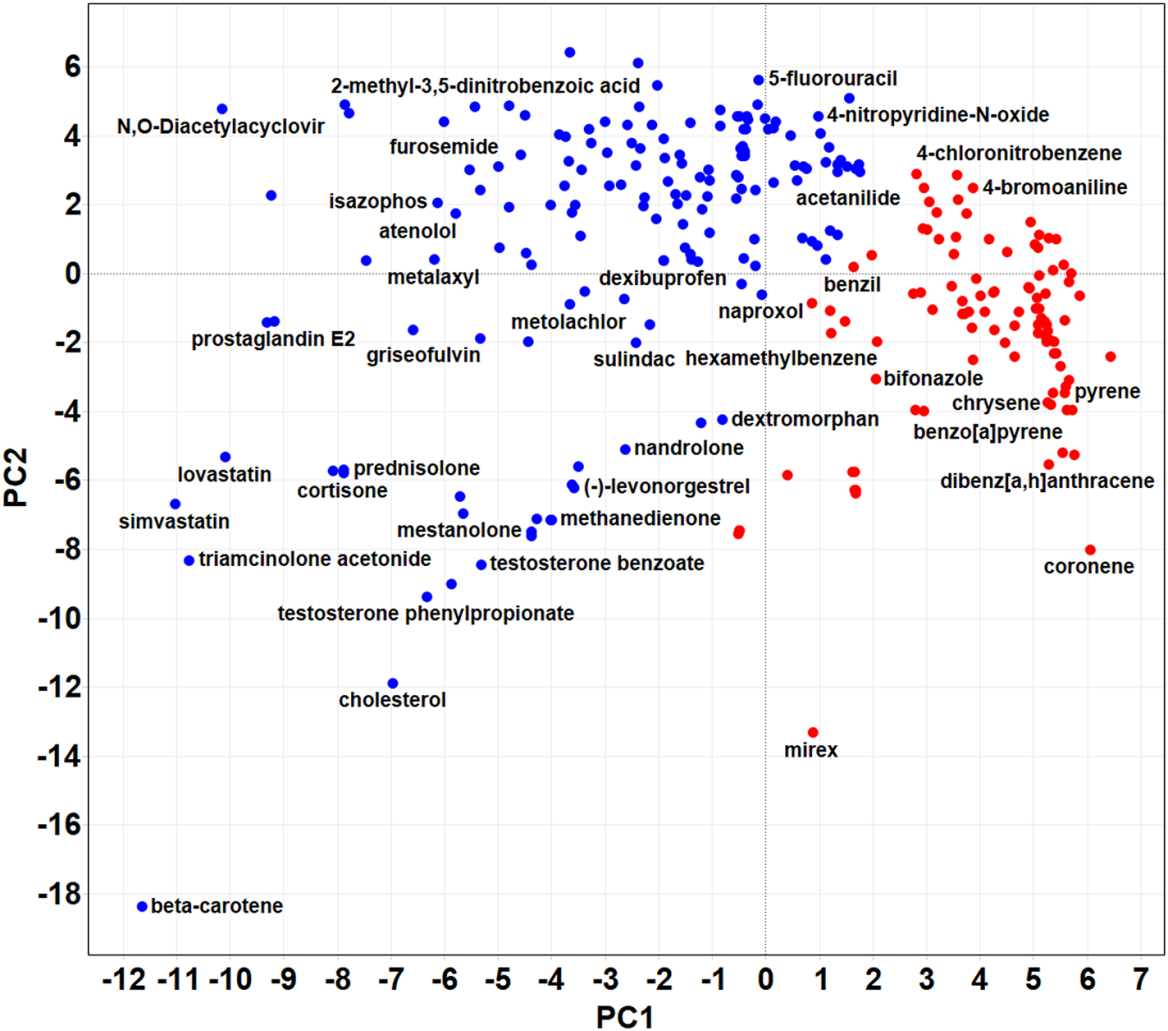
Chemical space of compounds naturally separate into two distinct clusters

Principal component analysis (using the *prcomp* function with *scale* *=* *T*) and cluster analysis was performed on the dataset of 259 compounds with 86 CDK descriptors using R. The optimal number of clusters was determined to be 2 by using silhouette analysis (using the *pam* function) on a series ranging from 2 to 20 clusters. The silhouettes had an average width of 0.74 for 2 clusters; almost double the next closest value [10]. The clusters are shown in Fig. 4 below with the x and y axes corresponding to the first and second principal components respectively. The first two principal components explain 36 % of the variance. The first cluster (red) is typified by compounds without hydrogen bond acceptors and with ALogP >1.56 and with TopoPSA <26.48; 128 out of 157 compounds match this criteria. The blue cluster is more chemically diverse than the red cluster but even so 75 of the 102 compounds have ALogP <1.56 and TopoPSA >26.48 and at least one hydrogen bond acceptor.

## Results and discussion

### Modeling

A Random Forest Model is a compilation of uncorrelated decision trees used to choose the best case among many. Our model used 86 variables in its calculation. In general, the less correlated that the variables are, the better the results that will occur from a random forest model. A higher strength of each individual tree also improves the accuracy of the final model—“The strength of each individual tree in the forest. A tree with a low error rate is a strong classifier. Increasing the strength of the individual trees decreases the forest error rate.” [11]. Using a random forest model allows us to get out-of-bag (OOB) estimates which are akin to cross-validation and are useful for estimating the performance of models created using small datasets.

Using Rajarshi Guha’s CDK Descriptor Calculator (v 1.4.6) [12], we calculated the CDK [13–15] descriptors for all the compounds in our refined data file, selecting the option to add explicit hydrogens. Once descriptors were calculated, we deleted all columns that had a zero standard deviation. Additional feature selection was performed by removing columns that were highly correlated (0.9 and above). Two compounds were removed as they had several “NA” values across multiple descriptors. This left us with a dataset of 259 1-octanol solubility values with 86 CDK descriptors.

**Fig. 5:**
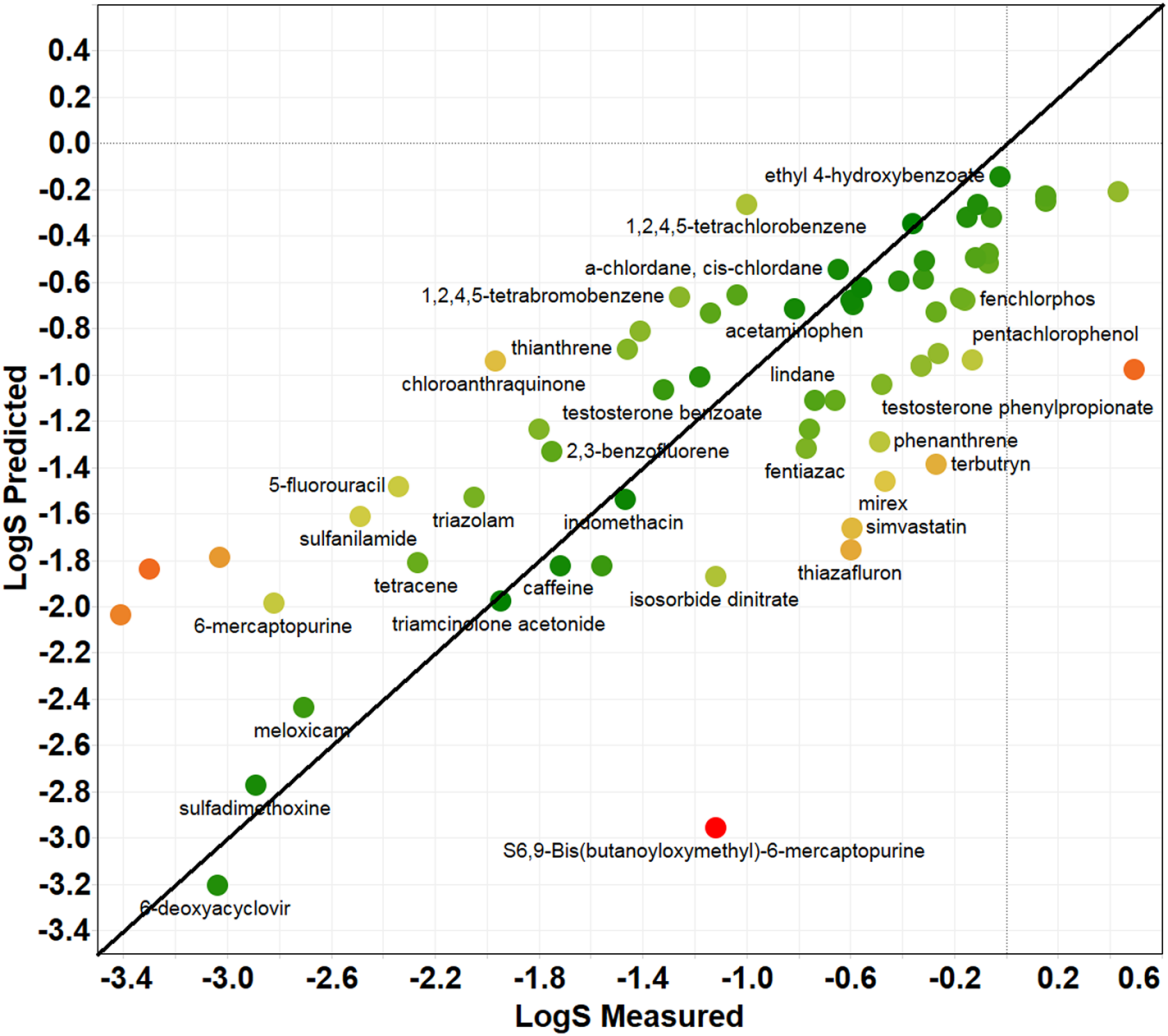
Predicted vs. measured solubility values for the randomly selected test-set coloured by AE

The dataset was then split randomly into training and test sets (75:25). Using the random forest model package (v 4.6-10) in R (v 3.1.2), we created a random forest model using our training set data. This model had an OOB R^2^ value of 0.63 and an OOB MSE of 0.38. This model was then used to predict the 1-octanol solubilities of the compounds in the test-set resulting in and R^2^ value of 0.54 and a MSE of 0.44, see Fig. 5. The performance statistics obtained when using the model to predict test-set solubilities are comparable to the OOB values. The fact that they are slightly smaller may be an artifact of the relatively small sizes of the training and test sets and the fact that we decided to doing a single taining-set/test-set split rather than use cross-validation.

One of the goals of our research was to provide for the community a useful web application that can be used to predict 1-octanol solubilities directly from structure. To accomplish this, we created a random forest model using the entire dataset. This model has an OOB R^2^ value of 0.66 and an OOB MSE of 0.34.

**Fig. 6:**
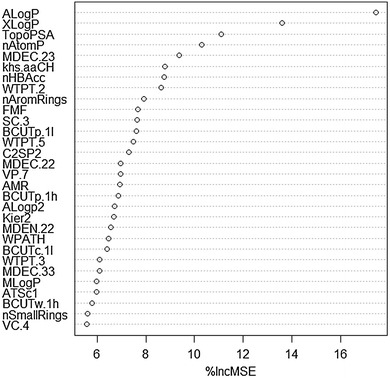
Random forest model variable importance

The following descriptors were identified as important: ALogP, XLogP, TopoPSA, nAtomP, MDEC.23, khs.aaCH, and nHBAcc, see Fig. 6, which correspond to two models for LogP, the predicted topological polar surface area, the number of atoms in the longest pi chain, the MDE topological descriptor, a Kier and Hall smarts descriptor, and the number of hydrogen bond acceptors respectively. It is not surprising that both ALogP and XLogP would be important in predicting 1-octanol solubility, though one would have assumed that one of these descriptors would have been removed during feature selection as being highly correlated with the other. Analyzing the correlation between these two descriptors, we see that they are correlated at 0.83 and they both survived as are cutoff was at 0.90. This further confirms the problems with current Open LogP descriptors implemented in the CDK [16].

We tried several other models using the same training set/test set split as above with no improvement in performance. A linear model (*lm*) using all 86 CDK descriptors had an R^2^ value of 0.24 and MSE of 0.88; A tuned (using tenfold cross validation) support vector machine (epsilon = 0.3, cost = 4.3) had an R^2^ value of 0.35 and MSE of 0.38; and an optimized (using the *train* command in the *caret* package) artificial neural network model (*nnet*) had an R^2^ value of 0.36 and MSE of 0.74. Thus the random forest model seems the best model for the current dataset.

**Fig. 7:**
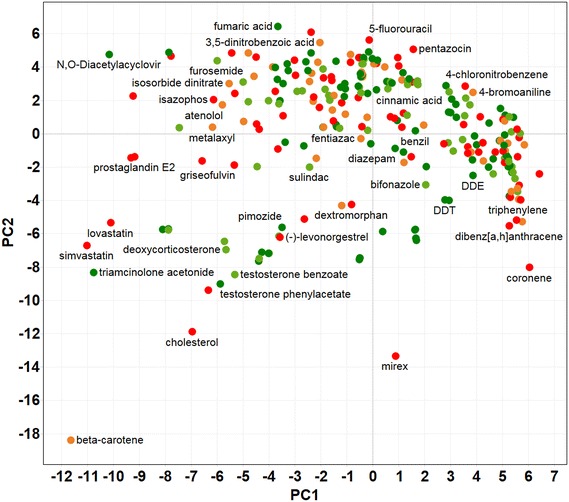
Training set chemical space where red indicates poor model performance

Previously published models only report the training set statistics, so in order to directly compare our model with previous models we used our full random forest model to predict the solubilities of the entire dataset, see Fig. 7. For the training set, the model has an R^2^ value of 0.94 and a MSE of 0.06. Abraham and Acree’s recommended Eq. (3), if all necessary descriptors are available, for estimations of log S_oct_ has a training set R^2^ value of 0.83 [5] which is lower than our value. Our model also does not require a measured melting point. This makes our model, even with the modest OOB R^2^ value of 0.66, superior to all others previously published.

In general, we expect the performance of our model to be better for compounds similar to those in the training set, apart from obvious outliers. However, there was no statistically significant performance differential between the interior and the periphery of the chemical space as has been found previously for other properties we have modeled using similar techniques [17]. We used the free-to-use DMax Chemistry Assistant Software [18] to help discover regions of the chemical space where our random forest model performs poorly (and conversely, well). Interestingly, the only statistically noteworthy (p ~ 0.1) finding is that the model performance is dependent upon the solubility values themselves; with the model performing well for compounds with solubility values over 0.01 M and performing poorly for compounds with solubility values less than 0.01 M. This suggests that the solubility data is comparatively not as reliable for compounds with solubility values less than 0.01 M and that using the model to predict solubilities of compounds that have low solubilities should be done with caution. No other statistically significant or noteworthy differences in model performance were found based on both physical properties and structure/scaffold.

The data collection, curation, and modeling were all performed under Open Notebook Science (ONS) conditions. Additional modeling details, including our R code, can be found on the Open Notebook page [19]. We have deployed our model as a Shiny application [20].

## Conclusions

We have developed a random forest model for 1-octanol solubility that has an OOB R^2^ value of 0.66 and an average absolute error of 0.34 that performs better than any other currently published model. Our model makes 1-octanol solubility predictions directly from structure without having to know the solute’s melting point or aqueous solubility. This makes our model the leading open model for predicting 1-octanol solubilities for a variety of applications.

## Additional file

10.1186/s13065-015-0131-2 1-octanol solubility values from the Open Notebook Science Challenge.

## Abbreviations

LFER: linear free energy relationship
CSID: chemspider ID
CDK: chemistry development kit
OOB: out-of-bag
ONS: open notebook science
MSE: mean squared error
AE: absolute error

## References

[1] Li A, Pinsuwan S, Yalkowsky SH (1995). Estimation of solubility of organic compounds in 1-octanol. Ind Eng Chem Res.

[2] Sepassi K, Yalkowsky SH (2006). Solubility prediction in octanol: a technical note. AAPS PharmSciTech.

[3] Raevsky OA, Perlovich GL, Schaper K-J (2007). Physicochemical properties/descriptors governing the solubility and partitioning of chemicals in water-solvent-gas systems. Part 2. Solubility in 1-octanol. SAR QSAR Environ Res.

[4] Admire B, Yalkowsky SH (2013). Predicting the octanol solubility of organic compounds. J Pharm Sci.

[5] Abraham MH, Acree WE (2014). The solubility of liquid and solid compounds in dry octan-1-ol. Chemosphere.

[6] Bradley J-C, Neylon C, Guha R, Williams A, Hooker B, Lang ASID, Friesen B, Bohinski T, Bulger D, Federici M, Hale J, Mancinelli J, Mirza K, Moritz M, Rein D, Tchakounte C, Truong H (2010). Open notebook science challenge: solubilities of organic compounds in organic solvents. Nat Precedings.

[7] Open Notebook Science. Wikipedia. Wikimedia Foundation. http://en.wikipedia.org/wiki/Open_notebook_science. Accessed 1 Sept 2015

[8] Bradley J-C, Guha R, Hooker B, Koch SJ, Lang ASID, Neylon C, Williams AJ (2015). Open Notebook Science Challenge Solubility Dataset. Figshare.

[9] Bradley J-C, Abraham MH, Acree WE, Lang ASID, Beck SN, Bulger DA, Clark EA, Condron LN, Costa ST, Curtin EM, Kurtu SB, Mangir MI, McBride MJ (2015). Determination of Abraham model solute descriptors for the monomeric and dimeric forms of trans-cinnamic acid using measured solubilities from the Open Notebook Science Challenge. Chem Cent J.

[10] Partitioning Around Medoids (PAM) Object. R Documentation. https://stat.ethz.ch/R-manual/R-devel/library/cluster/html/pam.object.html. Accessed 1 Sept 2015

[11] Breiman L, Cutler A. Random Forests. https://www.stat.berkeley.edu/~breiman/RandomForests/cc_home.htm. Accessed 1 Sept 2015

[12] Guha R. CDK Descriptor UI. https://github.com/rajarshi/cdkdescui. Accessed 1 Sept 2015

[13] The Chemistry Development Kit http://sourceforge.net/projects/cdk. Accessed 1 Sept 2015

[14] Steinbeck C, Han Y, Kuhn S, Horlacher O, Luttmann E, Willighagen E (2003). The Chemistry Development Kit (CDK): an open-source Java library for chemo-and bioinformatics. J Chem Inf Comput Sci.

[15] Steinbeck C, Hoppe C, Kuhn S, Floris M, Guha R, Willighagen EL (2006). Recent developments of the chemistry development kit (CDK)-an open-source java library for chemo-and bioinformatics. Curr Pharm Des.

[16] Guha R (2013) CDK and logP Values. So much to do, so little time. http://blog.rguha.net/?p=896. Accessed 1 Sept 2015

[17] Bradley J-C, Abraham MH, Acree WE, Lang ASID (2015). Predicting Abraham model solvent coefficients. Chem Cent J.

[18] DeGrave K (2010) DMax Chemistry Assitant. http://dtai-old.cs.kuleuven.be/ml/systems/dmax. Accessed 1 Sept 2015

[19] Buonaiuto MA, Lang ASID. Modeling the Solubility of Organic Compounds in 1-Octanol. ORU Open Notebook Science. Open Lab Notebook Page: http://oruopennotebookscience.wikispaces.com/CDK001. Accessed 1 Sept 2015

[20] Buonaiuto MA, Lang ASID (2015) Open 1-octanol solubility model. Shiny. http://michaeloru.shinyapps.io/Shiny. Accessed 1 Sept 2015

